# Evaluating a Novel AI Tool for Automated Measurement of the Aortic Root and Valve in Cardiac Magnetic Resonance Imaging

**DOI:** 10.7759/cureus.59647

**Published:** 2024-05-04

**Authors:** Jack Parker, James Coey, Tarek Alambrouk, Samuel M Lakey, Thomas Green, Alexander Brown, Ian Maxwell, David P Ripley

**Affiliations:** 1 Health and Life Sciences, Northumbria University, Newcastle upon Tyne, GBR; 2 Imaging, AIATELLA Oy, Helsinki, FIN; 3 Imaging, AIATELLA Ltd., Newcastle upon Tyne, GBR; 4 School of Medicine, St. George's University, Newcastle upon Tyne, GBR; 5 Cardiology, Northumbria Healthcare NHS Foundation Trust, Newcastle upon Tyne, GBR; 6 Faculty of Health Sciences and Wellbeing, University of Sunderland, Sunderland, GBR

**Keywords:** cardiovascular radiology, artificial intelligence in radiology, aortic root dilation, aortic valve disease, cardiac magnetic resonance (cmr), aortic root, aortic valve, artificial intelligence, interobserver variability, measurement accuracy

## Abstract

Objective

Evaluating an artificial intelligence (AI) tool (AIATELLA, version 1.0; AIATELLA Oy, Helsinki, Finland) in interpreting cardiac magnetic resonance (CMR) imaging to produce measurements of the aortic root and valve by comparison of accuracy and efficiency with that of three National Health Service (NHS) cardiologists.

Methods

AI-derived aortic root and valve measurements were recorded alongside manual measurements from three experienced NHS consultant cardiologists (CCs) over three separate sites in the northeast part of the United Kingdom. The study utilised a comprehensive dataset of CMR images, with the intraclass correlation coefficient (ICC) being the primary measure of concordance between the AI and the cardiologist assessments. Patient imaging was anonymised and blinded at the point of transfer to a secure data server.

Results

The study demonstrates a high level of concordance between AI assessment of the aortic root and valve with NHS cardiologists (ICC of 0.98). Notably, the AI delivered results in 2.6 seconds (+/- 0.532) compared to a mean of 334.5 seconds (+/- 61.9) by the cardiologists, a statistically significant improvement in efficiency without compromising accuracy.

Conclusion

AI's accuracy and speed of analysis suggest that it could be a valuable tool in cardiac diagnostics, addressing the challenges of time-consuming and variable clinician-based assessments. This research reinforces AI's role in optimising the patient journey and improving the efficiency of the diagnostic pathway.

## Introduction

Aortic pathologies, such as valve stenosis and root dilation, frequently progress without symptoms, posing significant risks of severe secondary complications if left undiagnosed and untreated. These complications include aneurysmal formation, aortic dissection and/or rupture [1,2,3]. In addition, these conditions are intricately associated with an increased incidence of cardiovascular comorbidities, such as chronic kidney disease and atrial fibrillation (27.3% and 15.9% of cases, respectively) [4,5]. Epidemiological data suggest a gradational increase in aortic pathologies with advancing age. The incidence of acute aortic dissection (AAD) in the UK is projected to increase from 3,892 in 2010 to 6,893 in 2050, and due to the high rate of mortality associated with aortic pathology, early detection is paramount, as is the regular surveillance of patients diagnosed with aortic root dilation that do not meet surgical criteria [5,6,7]. Cardiac magnetic resonance imaging (CMR) has emerged as an essential tool for the non-invasive evaluation and longitudinal monitoring of these conditions. Despite its efficacy, the manual interpretation of CMR data has significant associated time expenditure and susceptibility to interobserver variability [8], underscoring the necessity for more streamlined and reproducible diagnostic methodologies.

In recent years, artificial intelligence (AI) has emerged as a transformative technology in various fields, including healthcare and medical imaging [9]. AI, particularly machine learning and deep learning algorithms, have shown promising results in automating and enhancing the analysis of medical images [10]. These technologies can learn from large volumes of data, identify complex patterns and make predictions with high accuracy [11]. In the context of medical imaging, AI tools have demonstrated robust results in automating the detection of pathologies, such as lung nodules from chest imaging [12]. Other tools are now showing potential in providing a more comprehensive analysis of medical imaging, including diagnostic measurements [13], thereby reducing the time and effort of healthcare professionals spent on manual, rule-based tasks and minimising interobserver variability [14].

The present study introduces the novel AI tool ‘AIATELLA’ (version 1.0; AIATELLA Oy, Helsinki, Finland). This tool leverages deep learning algorithms to automate the measurement of the aortic root and valve, which are critical for diagnosing and monitoring aortic pathologies. AIATELLA has been trained on CMR data to accurately identify and measure the aortic root and valve. By comparing AIATELLA’s performance with conventional manual measurement techniques, this study aims to evaluate the potential of AI in enhancing the diagnosis and monitoring of aortic pathologies. This could represent a significant step forward in the field of medical imaging, potentially leading to earlier detection and treatment of these conditions.

## Materials and methods

Data collection

We identified a registry of patients who had undergone CMR imaging for the diagnosis or evaluation of aortic or cardiac disease with steady-state free precession (SSFP) cine sequences using two established MRI systems: Optima MR450w 1.5T (GE, Chicago, USA) and MAGNETOM Aera 1.5T (Siemens Healthineers, Erlangen, Germany). Thirty-five patients were selected from this registry adhering to the criteria in Table 1. Imaging series specific to the aortic root and valve were isolated. The resulting data were anonymised at source prior to transfer to the AI developer. This study received ethical approval from the Northumbria University Ethics Committee under project number 6822. All procedures performed in the study were in accordance with the ethical standards of the institutional and/or national research committee and with the 1964 Helsinki Declaration and its later amendments or comparable ethical standards.

**Table 1:** Inclusion and exclusion criteria for imaging data

Inclusion criteria	Exclusion criteria
≥18 years old	<18 years
Consent obtained for use of de-identified imaging	Bicuspid valve
≥1.5T MRI	Known congenital pathology
	Previous aortic surgery

Clinical image analysis

The cine-CMR images of the aortic root and valve were analysed by three Level 3 SCMR/EuroCMR-accredited National Health Service (NHS) consultant cardiologists (CCs) during routine workflow using cvi42® software (Calgary, Canada) to produce the ground truth. A timer was used to record the time to measure. 

Quantitative evaluation of the aortic root and valve was carried out using static images from aortic valve cine sequences that best represented the peak systolic and end diastolic phases of the cardiac cycle, in accordance with European Society of Cardiology (ESC)/European Association for Cardio-Thoracic Surgery (EACTS) guidelines for the management of valvular heart disease [15]. For the purpose of this study, a singular measurement of the aortic root area was recorded at the level of the sinuses of Valsalva (SoV). The aortic valve area (AVA) is utilised as a reliable indicator of aortic valve stenosis using CMR, with <1 cm^2^ indicative of severe stenosis [16]. In this study, an AVA measurement was obtained during the peak systole, at the aortic annulus. An additional method for assessing the aortic root size, as recommended in [17], is to take three linear measurements between valve cusps and three additional linear measurements from each cusp to the adjacent valve commissure, at the level of the SoV. These measurements were obtained during the cardiac cycle's systolic and diastolic phases. The acquisition of all morphometric data by the cardiologists was through the use of cvi42® software (Circle Cardiovascular Imaging Inc., Calgary, Canada), with the duration of measurement time self-documented by the examining observers.

AI image analysis

**Figure 1:**
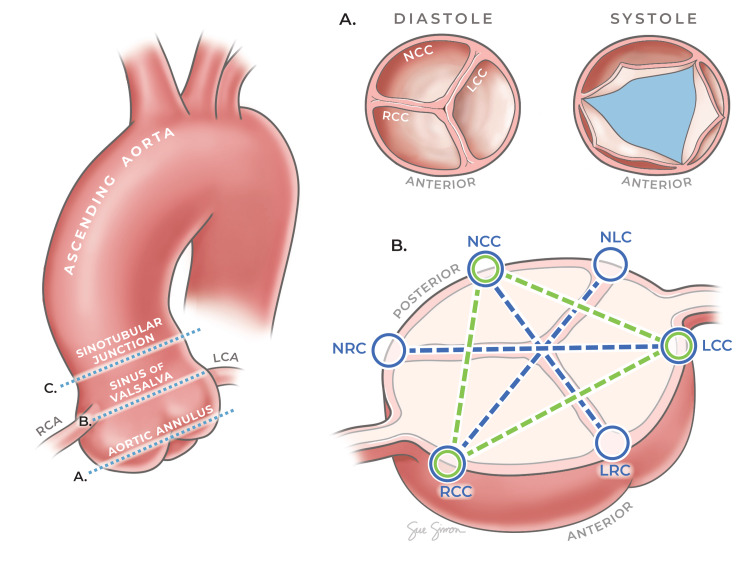
Anatomical cross-sections and measurements of the aortic root and valve This figure provides a comprehensive view of the aortic root, highlighting the levels of the aortic annulus, sinuses of Valsalva (SoV) and sinotubular junction (STJ). The main figure (left) illustrates the overall structure and the specific levels where measurements were taken. The sub-figures (right) provide detailed cross-sectional views at the aortic annulus and SoV levels, indicating the exact points of measurement. LCC: left coronary cusp, NCC: non-coronary cusp, RCC: right coronary cusp, LRC: left-right coronary commissure, NRC: non-coronary-right commissure, NLC: non-coronary-left commissure Printed with permission from Sue Simon, MS, CMI © CC-BY-ND 2024

The anonymised imaging transferred to the AI developer was immediately and randomly assorted to training and validation subsets in a 4:1 ratio. Data storage and analysis by 'AIATELLA' is hosted on the Microsoft Azure Cloud. Training of the AI model first requires the annotation of crucial anatomical landmarks and the valve lumen and root area. The training phase in this study utilised model parameters, such as weight adjustments and feature extraction, in an iterative process. The remaining 20% of the dataset, unseen by the model during its training phase, constituted the validation dataset. This subset functioned as an independent benchmark to assess the trained model's accuracy in measuring new data compared to the assessment of three cardiologists. The time taken to analyse these datasets by the 'AIATELLA' algorithm was also recorded independently. Figure 1 provides an illustration of the aortic root and the levels at which measurements were obtained. 

Statistical analysis

The study employed IBM SPSS Statistics for Windows (IBM Corp., Armonk, NY) for statistical analysis, utilising descriptive statistics and ANOVA to compare the measurements across groups. This analysis aimed to rigorously evaluate the performance differences between the cardiologists' assessments and the AI's outputs.

## Results

**Figure 2:**
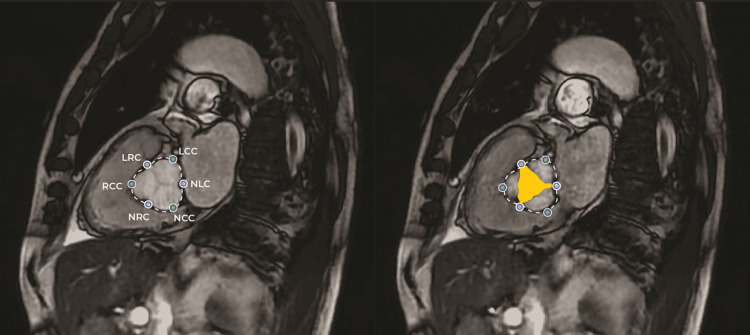
CMR imaging with AI-generated measurements and landmarks This figure presents an example of the cardiovascular magnetic resonance (CMR) imaging used in this study, featuring AI-generated measurements. The left panel illustrates the diastolic phase of the cardiac cycle, showcasing AI-identified anatomical landmarks including the left coronary cusp (LCC), non-coronary cusp (NCC), right coronary cusp (RCC), left-right coronary commissure (LRC), non-coronary-right commissure (NRC), and non-coronary-left commissure (NLC). The right panel depicts the systolic phase of the cardiac cycle, highlighting both the AI-detected landmarks and the area measurement of the aortic valve lumen, represented in yellow.

The efficacy of the AI tool 'AIATELLA' was evaluated by comparing it with the expertise of three CCs in measuring aortic root and valve structures. An example of the AI analysis is presented in Figure 2.

As depicted in Table 2, the statistical analysis demonstrated substantial agreement between the AI-generated measurements and those conducted by the cardiologists, supporting the AI's capacity for accurate CMR analysis. Interclass correlation coefficient (ICC) values indicate statistically significant congruity across all observers. When considering interobserver variability, the measurements of the human observers showed a higher variance compared to AIATELLA (humans: ICC systole range 0.912-0.987, ICC diastole range 0.902-0.986), whereas the AI tool showed a more precise range (AIATELLA: ICC systole range 0.9-0.979, ICC diastole range 0.962-0.979).

**Table 2:** Agreement between observers This table shows the intraclass correlation coefficients (ICCs) demonstrating agreement levels between the human observers (CC1, CC2 and CC3) and all observers combined (CC1, CC2, CC3 and AIATELLA) for both the systole and diastole phases. The ICC values, given with 95% confidence intervals (CI), provide a measure of the reliability of measurements taken by the observers. The ICC values indicate high reliability in measurements across both phases. CC: consultant cardiologist

	Image phase	
Observer	Systole	Diastole
Human only	0.972 (0.912-0.987)	0.969 (0.902-0.986)
Combined	0.959 (0.9-0.979)	0.962 (0.915-0.979)

The speed of the AI tool was significant; the mean time to complete measurement was 2.6 seconds (+/- 0.532) compared to a mean of 334.5 seconds (+/- 61.9) by the cardiologists, as shown in Table 3. 

**Table 3:** Time taken to measure This table outlines the average time and standard deviation required by each observer to complete the measurements. It highlights the efficiency of the AI tool 'AIATELLA', which significantly outperforms human observers in speed, with a much lower standard deviation, suggesting a consistent performance. DNR: did not report

Observer	Mean time (seconds)	Standard deviation
CC1	DNR	N/A
CC2	368	83.074
CC3	301	40.704
AIATELLA	2.61	0.532

## Discussion

This research critically assessed the AI tool 'AIATELLA' for aortic root and valve analysis, focusing on its accuracy, efficiency and reliability compared to standard practices in cardiovascular care. This study highlights the potential of AI to supplement healthcare professionals in the quantitative evaluation of CMR imaging. The results demonstrated substantial agreement between AI-generated measurements and those conducted by cardiologists, with the AI tool showing notable efficiency and consistency. Here is a structured discussion based on these results: 

Requirements of measurement in cardiovascular assessment

Accurate measurement in cardiovascular assessment is paramount for effective diagnosis and treatment planning [18]. This typically involves the evaluation of various cardiac structures, including the aortic root and valve, and their functional parameters across different phases of the cardiac cycle. These measurements provide critical insights into the patient’s cardiovascular health and guide therapeutic interventions.

Limitations of manual methods

However, manual methods of cardiovascular measurement, typically conducted by human observers, have inherent limitations [19,20]. First, these methods are time-consuming and require a high level of expertise. This can lead to bottlenecks in high-volume clinical environments and may delay diagnosis and treatment.

Second, manual measurements are subject to inter-observer and intra-observer variability. Different observers may produce varying measurements for the same anatomical structure, and even the same observer may not produce identical measurements at different times. This variability can impact the consistency of diagnoses and limit the reliability of longitudinal patient monitoring [21,22].

Lastly, manual methods may not fully capture the complex, three-dimensional nature of cardiac structures in certain disease states, such as a highly calcified valve. This can lead to oversimplification of these structures and potential inaccuracies in measurement [23].

The integration of AI tools, such as the ‘AIATELLA’ tool assessed in this study, can help to overcome these limitations. The findings from this study underscore the potential to enhance the precision and efficiency of CMR analysis. The high degree of consistency, as indicated by near-perfect ICC values, suggests that the AI tool can match, if not exceed, the measurement accuracy typically associated with expert human observers. Such an outcome is significant for clinical settings, where the accuracy of aortic root and valve measurements is crucial for diagnosis, treatment planning and monitoring of cardiac conditions [24].

Efficiency and error reduction

The AI tool completed measurements over 100 times faster than CCs utilising current best practices (AI mean 2.6 seconds +/- 0.532, cardiologist mean 334.5 seconds +/- 61.9). This speed, while maintaining accuracy, could positively impact clinical workflows, allowing for real-time analysis and immediate decision-making in patient care. Reducing interobserver variability is particularly relevant in ensuring diagnosis consistency, a common challenge in medical imaging where subjective interpretations can lead to outcome variability [19,21]. 

The complementary role of AI tools in the diagnostic process

While AI tools may be more accurate than humans at producing measurements, the diagnostic process involves not only the accurate measurement of anatomical structures but also the interpretation of these measurements in the context of the patient’s overall health status, medical history and presenting symptoms [25]. Therefore, AI tools, such as the one assessed in this study, hold great potential in complementing clinicians in the diagnostic process. By automating measurements, these tools can free up valuable time for clinicians, allowing them to focus on interpreting imaging holistically.

AI tools can enhance this process by providing precise and consistent measurements quickly and efficiently [26]. This allows clinicians to dedicate more time to the overall clinical correlation of the case, integrating the AI-generated measurements with other relevant clinical information to arrive at a comprehensive and accurate diagnosis [27].

In this way, AI tools do not replace clinicians but rather serve as valuable aids in the diagnostic process. They combine the strengths of AI precision and speed with the invaluable clinical judgment and experience of healthcare professionals, leading to a more effective and efficient diagnostic process [26,27].

The integration of AI, such as the 'AIATELLA' tool, could be especially beneficial in high-volume clinical environments, where the demand for imaging studies often exceeds the capacity of specialist staff. In addition, the implementation in clinical environments with limited access to cardiovascular radiology expertise could provide benefits in healthcare accessibility and reduced dependency upon specialised clinicians, which are in short supply globally [28]. 

Limitations and future directions 

While the results presented are promising, it is important to acknowledge the study's limitations. A relatively small sample size was utilised, which may limit the generalisability of our findings across broader patient populations and varied pathological conditions. The AIATELLA tool must be validated with larger, more diverse cohorts and additional anatomical sites to ensure applicability in a broader clinical context [19]. Future research should also explore integrating AI tools into clinical workflows, assessing the impact on patient outcomes and healthcare resource utilisation. 

Furthermore, the potential for AI to complement the expertise of clinicians suggests a paradigm shift towards a hybrid model of patient care, where AI tools serve as an extension of the clinical team. This model would leverage the speed and consistency of AI while retaining the invaluable clinical judgment and experience of healthcare professionals in care delivery.

## Conclusions

This study indicates that AI tools, such as 'AIATELLA', can improve the efficiency and consistency of measurement in medical imaging. By reducing measurement times and maintaining a high level of accuracy, AI could assist in improving the overall efficiency of diagnostic pathways by complementing existing workflows. Incorporating AI tools into clinical practice necessitates a mindful review of its limitations and continuous assessment to fine-tune its application in conjunction with human expertise.
